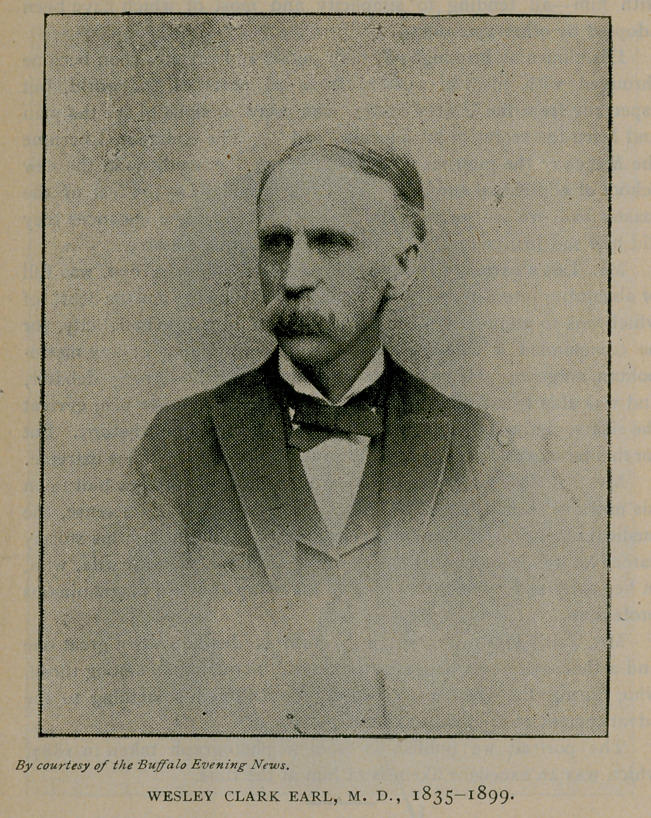# Dr. Wesley Clark Earl

**Published:** 1899-07

**Authors:** 


Dr. Wesley Clark Earl, of Buffalo, died at his residence, 147 Lafayette avenue, early Monday morning, June 19, 1899, aged 64 years. He was born January 13, 1835, at Mount Holly, Rutland County, Vermont, where he passed a portion of his early life. He began the study of medicine with Dr. M. S. Kittinger, of Lockport, entered the University of Buffalo and graduated from

Bellevue Hospital Medical College in 1864. Soon after graduation he was appointed acting assistant surgeon in the United States Army during the civil war and was first assigned to duty at Fort Schuyler. At a later period his assignment was changed to Elmira,

*By courtesy of the Buffalo Evening News.*

WESLEY CLARK EARL, M. D., 1835–1899.

N. Y., where he served in caring for confederate prisoners under Dr. William C. Wey, who was surgeon in charge.

After the war Dr. Earl located at Pekin, Niagara County, New York, where he conducted the practice of medicine for nine years. He removed to Buffalo in 1874, and here continued in practice until his death.

Dr. Earl was a representative of the best type of that somewhat underestimated, if not forgotten, element in the profession of medicine known as the family doctor. He was kind, faithful, skilful and upright. He acquired a large following in that section of the city where he was located and his numerous families were very much attached to him.

Dr. Earl for many years was a trustee of the Riverside Methodist Church. He was a member of Occidental Lodge, F. & A. M., a member of the Avonian Society and the Royal Templars of Temperance. He was also an examining physician for the A. O. U. W. and a member of the Society of Vermonters. A widow and one child, Mrs. Thomas M. Heard, Jr., survive him.

The funeral services were held at the Riverside M. E. Church and were numerously attended by his friends and acquaintances. The honorary bearers were Drs. S. S. Green, Henry R. Hopkins, A. A. Hubbell, S. W. Wetmore, and C. C. Wyckoff of Buffalo, and Dr. M. S. Kittinger, of Lockport. The active bearers were Hon. Henry W. Hill, Capt. M. M. Drake, Dr. John C. Thompson, Dr. Benjamin Lothrop, A. C. Abrams and M. A. Root. The burial was at Forest Lawn.

## Figures and Tables

**Figure f1:**